# Heterodimer of A2A and Oxytocin Receptors Regulating Glutamate Release in Adult Striatal Astrocytes

**DOI:** 10.3390/ijms23042326

**Published:** 2022-02-19

**Authors:** Sarah Amato, Monica Averna, Diego Guidolin, Marco Pedrazzi, Simone Pelassa, Michela Capraro, Mario Passalacqua, Matteo Bozzo, Elena Gatta, Deanna Anderlini, Guido Maura, Luigi F. Agnati, Chiara Cervetto, Manuela Marcoli

**Affiliations:** 1 Department of Pharmacy, Section of Pharmacology and Toxicology, University of Genova, Viale Cembrano 4, 16148 Genova, Italy; amato@difar.unige.it (S.A.); simonepelassa@gaslini.org (S.P.); maura@pharmatox.unige.it (G.M.); 2 Department of Experimental Medicine, Section of Biochemistry, University of Genova, Viale Benedetto XV 1, 16132 Genova, Italy; monica.averna@unige.it (M.A.); marco.pedrazzi@unige.it (M.P.); capraro36@gmail.com (M.C.); mario.passalacqua@unige.it (M.P.); 3 Department of Neuroscience, University of Padova, Via Gabelli 63, 35122 Padova, Italy; diego.guidolin@unipd.it; 4 Italian Institute of Biostructures and Biosystems, Viale delle Medaglie d’Oro 305, 00136 Roma, Italy; 5 Department of Earth, Environment and Life Sciences, University of Genova, Viale Benedetto XV 5, 16132 Genova, Italy; matteo.bozzo@edu.unige.it; 6 DIFILAB, Department of Physics, University of Genoa, Via Dodecaneso 33, 16146 Genova, Italy; gatta@fisica.unige.it; 7 Centre for Sensorimotor Performance, The University of Queensland, Brisbane, Blair Drive, St. Lucia, QLD 4067, Australia; deanna.anderlini@uqconnect.edu.au; 8 Department of Biomedical, Metabolic Sciences and Neuroscience, University of Modena and Reggio Emilia, Via Campi 287, 41125 Modena, Italy; luigi.agnati@gmail.com; 9 Center of Excellence for Biomedical Research, University of Genova, Viale Benedetto XV 9, 16132 Genova, Italy

**Keywords:** heterodimers, astrocyte processes, striatum, neuroglia, glutamate, rat, molecular modeling

## Abstract

Background: Roles of astrocytes in the modulatory effects of oxytocin (OT) in central nervous system are increasingly considered. Nevertheless, OT effects on gliotransmitter release have been neglected. Methods: In purified astrocyte processes from adult rat striatum, we assessed OT receptor (OTR) and adenosine A2A receptor expression by confocal analysis. The effects of receptors activation on glutamate release from the processes were evaluated; A2A-OTR heteromerization was assessed by co-immunoprecipitation and PLA. Structure of the possible heterodimer of A2A and OT receptors was estimated by a bioinformatic approach. Results: Both A2A and OT receptors were expressed on the same astrocyte processes. Evidence for A2A-OTR receptor-receptor interaction was obtained by measuring the release of glutamate: OT inhibited the evoked glutamate release, while activation of A2A receptors, per se ineffective, abolished the OT effect. Biochemical and biophysical evidence for A2A-OTR heterodimers on striatal astrocytes was also obtained. The residues in the transmembrane domains 4 and 5 of both receptors are predicted to be mainly involved in the heteromerization. Conclusions: When considering effects of OT in striatum, modulation of glutamate release from the astrocyte processes and of glutamatergic synapse functioning, and the interaction with A2A receptors on the astrocyte processes should be taken into consideration.

## 1. Introduction

Oxytocin (OT) is a peptide hormone with a major role in mammalian behavior and health and functioning as a “stress-coping molecule” [1]. OT is recognized to play wide modulatory effects in the central nervous system of mammals, regulating not only pair bonding, sexual and maternal behavior, but also a huge wide pattern of behaviors such as social behavior, motivation [2], modulation of emotional states [3], stable attachment formation and stress coping by shifting processing from novelty and reward seeking to appreciation of familiarity [4], anxiety, trust, sociability, sleep-awake cycle [5], food intake or drug abuse [6,7,8]. Such wide effects have been attributed to activation of OT receptors mainly localized on excitatory or inhibitory neurons, at somatodendritic or at presynaptic regions, accounting for regulation of neuronal excitability [9,10] and neurotransmitter release [10]. OT effects on social-emotional behavior and potential effectiveness in autism spectrum disorders, in dysfunction of the emotional networks or drug abuse disorder have been hypothesized via its actions at striatum [11,12,13].

Astrocytes have been suggested to be involved in OT effects on amygdala [3], hypothalamus [14,15] and hippocampus [16]. In the amygdala, a subpopulation of astrocytes expressing OT receptors was found to mediate the OT anxiolytic and positive reinforcement effects [3]. Evidence for the involvement of astrocytes in OT signaling challenges the long-held dogma that OT acts exclusively on neurons and highlights how astrocytes are essential components for modulation of neuronal activity and emotional states [3]. Indeed, evidence has accumulated supporting a direct involvement of astrocytes in diverse aspects of behavior including cognition, emotion, and motor and sensory processing [17].

Interestingly, it has been reported that OT secretion during suckling reduces glial fibrillary acidic protein (GFAP) expression and causes retraction of astrocytic processes in supraoptic nucleus; GFAP’s plasticity dynamically reflects OT neuronal activity, and in turn modulates OT neuronal activity by changing astrocyte morphology and glutamate metabolism in astrocytes [14,15]. OT was also reported to affect the perisynaptic astrocyte processes (PAPs) motility in cultured astrocytes and hippocampal slices [16]. The ability of PAPs to retract and regulate coverage of the synapse therefore reducing synapse efficiency [18] and regulating the ability of neurotransmitters to escape from the synapse and diffuse to long-distance targets [19,20,21,22], may be crucial to the balance and integration of wiring and volume transmission [23,24,25,26] in the brain integrative functions. Notably, OT was found to be released mostly non-synaptically and act mainly through volume transmission by diffusing at distance from the sites of release [27,28]. Therefore, the reports suggesting the OT ability to control astrocytic coverage of the synapses [14,15,16] could be a step-ahead to understand its wide modulatory effects.

The astrocytic processes, carrying crucial functions at the tripartite synapses such as clearance of extracellular glutamate through excitatory amino acid transporters EAAT [29,30], clearance of K^+^ and modulation of network function [31] besides regulation of synapse coverage, can release gliotransmitters in a vesicular Ca^2+^-dependent manner [32]. As described, the astrocyte processes are involved in a bidirectional neuron-astrocyte communication, responding to neuronal activity, regulating synapse efficiency, transmitter diffusion and releasing signals that can affect neuron function at the synapses or as long-distance signals [33,34,35]. At striatal level, astrocytic glutamate release and uptake are suggested to influence the efficacy of glutamatergic synaptic transmission also on a long-term scale, and diversity of astrocytes is supposed to contribute to the diversity of presynaptic modifications involving striatal glutamatergic dysfunction in pathological conditions [36]. Indeed, in the dorsal striatum distinct subpopulations of astrocytes—in response to cortical stimulation [37]—release glutamate that activates N-methyl-D-aspartate (NMDA) receptors on specific medium spiny neurons and metabotropic glutamate receptors at distal synapses [38]. In ventral striatum astrocyte-neuron signaling is well established; astrocytes respond to neurotransmitters with Ca^2+^ increases and release of gliotransmitters—including glutamate and ATP/adenosine—then modulating neuronal activity and synaptic transmission [39].

Here we investigated the effects of OT on the release of the gliotransmitter glutamate from striatal astrocytes by using a purified preparation of the astrocyte processes obtained from adult rat striatum. Furthermore, we investigated if OT effects can be modulated by A2A receptor activation through a receptor-receptor interaction (RRI). The ability of native striatal astrocytic A2A and OT receptors (OTR) to heteromerize was assessed by co-immunoprecipitation and proximity ligation assay (PLA). To predict the possible structure of the A2A-OTR heterodimer and the residues mainly involved in the heteromerization a molecular modeling approach was followed.

## 2. Results

### 2.1. Oxytocin Receptor Activation Inhibits the 4-AP-Evoked Release of Glutamate from Striatal Astrocyte Processes and Ca^2+^ Influx into the Processes

**Figure 1:**
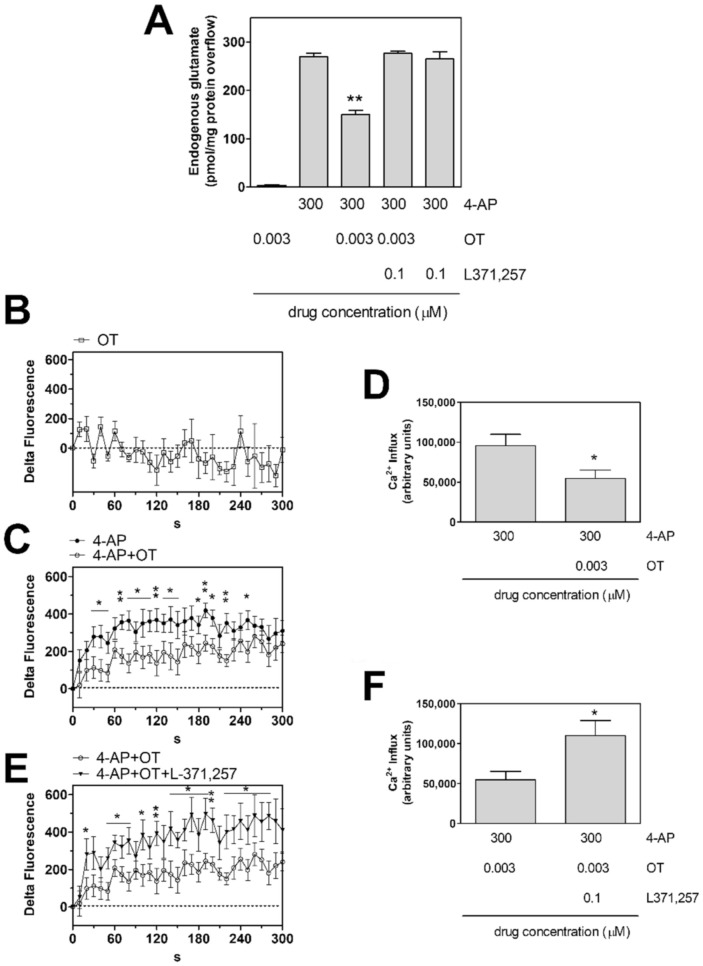
Endogenous glutamate release and intracellular calcium increase in response to 4-AP induced depolarization in striatal gliosomes. Modulation by OT. (**A**) Inhibition by OT 3 nM of the 4-AP-evoked endogenous glutamate efflux. Bars represent the overflow of the glutamate release, expressed as pmol/mg protein in 3 min sample, in the presence of the drugs at the concentrations indicated. 4AP was added (6 min) during superfusion; OT was added together with 4-AP; the OT receptor antagonist L371,257 was added 8 min before the agonist. Other experimental details in Materials and Methods. Data are means ± SEM (bars) of *n* = 3–6 independent experiments. ** *p* < 0.01 compared with the effect of 4-AP, according to Kruskal-Wallis two tailed test and multiple comparison analysis. (**B**–**F**) CG-loaded gliosomes were treated with 3 nM OT (**B**), or with 300 µM 4-AP in the absence (●) or presence (○) of 3 nM OT (**C**,**D**), or with 300 µM 4-AP + 3 nM OT in the absence (○) or presence (▼) of the OT receptor antagonist L371,257 (**E**,**F**) for the indicated time at 37 °C. CG-dependent fluorescence was monitored every 10 s from 0 to 300 s. [Ca^2+^]_i_ increase is expressed as “Delta Fluorescence”, which is the difference between the CG-dependent fluorescence of the stimulated samples and the ones of the vehicle-treated samples, both measured at each recording time and subtracted by the one measured at the starting time. The areas reported in (**D**,**F**) were quantified to estimate the calcium influxes after 300 s. Data are means ± SEM from three (**B**), nine and eight ((**C**,**D**), ● and ○, respectively), and eight and five ((**E**,**F**), ○ and ▼, respectively) experiments in duplicate. * *p* < 0.05 and ** *p* < 0.01, according to Mann-Whitney test. 4-AP, 4-aminopyridine; CG, Calcium Green™-1 AM; L371,257, 1-[4-[(1-Acetyl-4-piperidinyl)oxy]-2methoxybenzoyl]-4-(2-oxo-2H-3,1-benzoxazin-1(4H)-yl)piperidine; OT, oxytocin.

Endogenous glutamate release from astrocytic processes in superfusion was studied. The basal endogenous glutamate outflow in the first two fractions collected from the processes amounted to 87.0 ± 4.9 pmol/mg protein min (*n* = 19). 4-AP (300 µM) increased the endogenous glutamate efflux (4-AP 300 µM evoked overflow: 269.1 ± 5.6 9 pmol/mg protein; *n* = 19). The capability of 4-AP to evoke efflux of endogenous glutamate (Supplementary Figure S1A, Figure 1A and Figure 4A) was consistent with the findings obtained by measuring the 4-AP-evoked efflux of the glutamate analogue [^3^H]D-aspartate, which also indicated that 4-AP evoked exocytotic release of the gliotransmitter, dependent on Ca^2+^ entry [40]. Consistently, by measuring [Ca^2+^]_i_ changes, we found that 4-AP evoked increase in [Ca^2+^]_i_ in the rat striatal astrocyte processes (Figure S1B, Figure 1C–F and Figure 4B,C). Preliminary experiments have shown that OT 3 nM could inhibit the 4-AP evoked efflux of endogenous glutamate as well as the evoked increase in [Ca^2+^]_i_, while OT was ineffective at 1 nM or 10 nM (Supplementary Figure S1). The 3 nM concentration was then chosen to further investigate on OT effects on striatal astrocytic processes. Notably, the effective concentration of OT (3 nM) was the same which was found effective in the D2-OTR heterodimer on striatal neurons [11]. OT (3 nM) inhibited the [Ca^2+^]_i_ response to 4-AP in striatal processes (Figure 1C,D) and the glutamate releasing effect of 4-AP from the processes (Figure 1A). The OTR antagonist L-371,257 (0.1 µM), per se ineffective on the 4-AP-evoked glutamate release or [Ca^2+^]_i_, prevented the OT (3 nM) inhibition of the 4-AP-evoked [Ca^2+^]_i_ response and glutamate release (Figure 1A,E,F). OT (3 nM) had no effect on the basal [Ca^2+^]_i_ levels in gliosomes (Figure 1B), as well as on the basal glutamate efflux (Figure 1A).

Collectively, the findings indicate that 4-AP elicited Ca^2+^ entry in striatal astrocyte processes and evoked release of glutamate from the processes. Also, activation of OT receptors was able to inhibit both the 4-AP-evoked Ca^2+^ entry and the glutamate release from the processes.

### 2.2. Both A2A and OT Receptors Are Co-Localized on Striatal Astrocytic Processes

**Figure 2:**
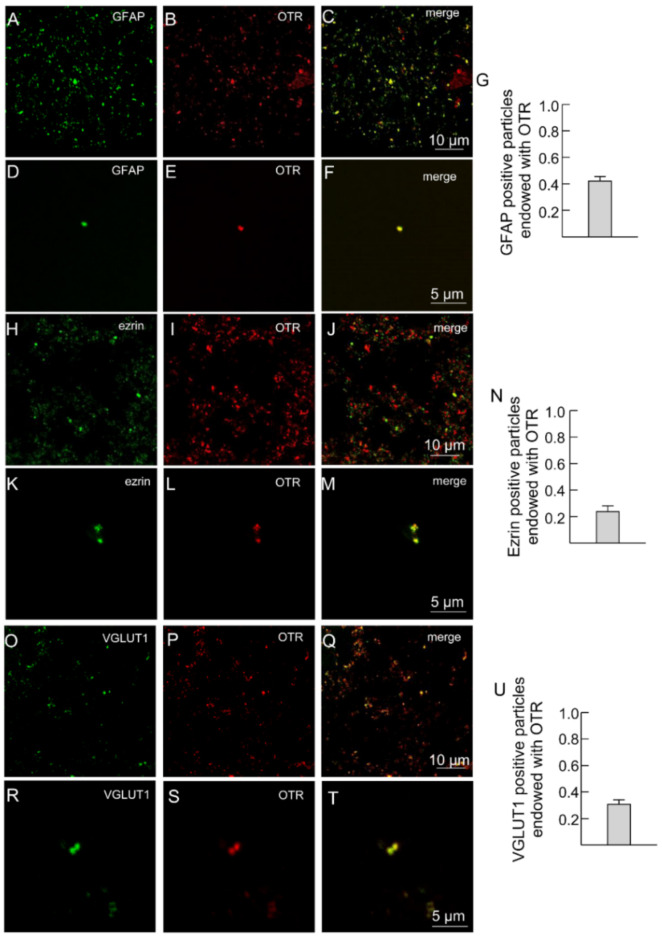
Striatal gliosomes express OT receptors. (**A**–**G**) Confocal images showing co-localization of OTR with GFAP. Double immunofluorescence labeling in striatal gliosomes with antibody against the astrocyte marker GFAP ((**A**,**D**), green), the OT receptor ((**B**,**E**), red). Merge images showing co-expression of the markers (**C**,**F**) and the bar (**G**) indicates the overlapping ratio expressed as mean ± SEM of 15 fields from three different preparations. Note a single GFAP process expressing OT receptors (**D**–**F**). (**H**–**N**) Confocal images showing co-localization of OT with ezrin. Double immunofluorescence labeling in striatal gliosomes with antibody against the astrocytic process Ezrin ((**H**,**K**), green) and the OT receptor ((**I**,**L**), red fluorescence). Merge images showing co-expression of the markers (**J**,**M**) and the bar (**N**) indicates the overlapping ratio expressed as mean ± SEM of 19 fields from three different preparations. Note a single ezrin-positive process expressing OT receptors (**K**–**M**). (**O**–**U**) Confocal images showing co-localization of OTR with VGLUT1. Double immunofluorescence labeling in striatal gliosomes with antibody against the VGLUT1 ((**O**,**R**), green) and the OT receptor ((**P**,**S**), red). Merge images showing co-expression of the markers (**Q**,**T**) and the bar (**U**) indicates the overlapping ratio expressed as mean ± SEM of 15 fields from three different preparations. Note a single VGLUT1-positive process expressing OT receptors (**R**–**T**).

Astrocytic processes proved to be positive for the astrocytic fibrillary acidic protein (GFAP) markers, for ezrin which is the preferential fine PAPs marker and for the vesicular glutamate transporter type 1 (VGLUT1). All were labeled with anti-OTR (Figure 2A–T).

In the figure representative fields are shown. Scale bars are indicated in the figures. GFAP, glial fibrillary acidic protein; OTR, oxytocin receptor; VGLUT1, vesicular glutamate transporter type 1.

**Figure 3:**
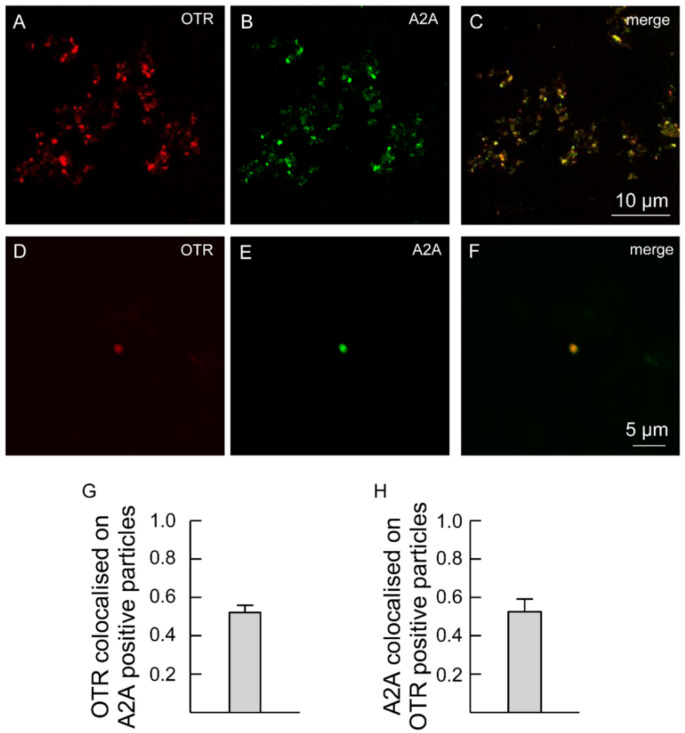
Striatal gliosomes co-express A2A and OT receptors. (**A**–**F**) Confocal images showing co-localization of A2A with OT receptors. Double immunofluorescence labeling in striatal gliosomes with antibody against the OT receptor ((**A**,**D**), red) and the adenosine A2A receptor ((**B**,**E**), green fluorescence). Merge images showing co-expression of the markers (**C**,**F**) and the bars (**G**,**H**) indicate the overlapping ratio expressed as mean ± SEM of 30 fields from four different preparations. Note a single gliosome expressing OTR and A2A (**D**–**F**). OTR, oxytocin receptor; A2A, adenosine receptor.

Moreover, the processes were labeled with anti-A2A antibodies (Figure 3), indicating that the processes express both A2A and OT receptors (Figure 3A–H). Single astrocyte processes expressing both A2A and OTR are shown at higher magnification in Figure 3D–F.

### 2.3. Activation of A2A Receptor Prevents the Oxytocin Inhibition of Glutamate Release from Striatal Astrocytic Processes

**Figure 4:**
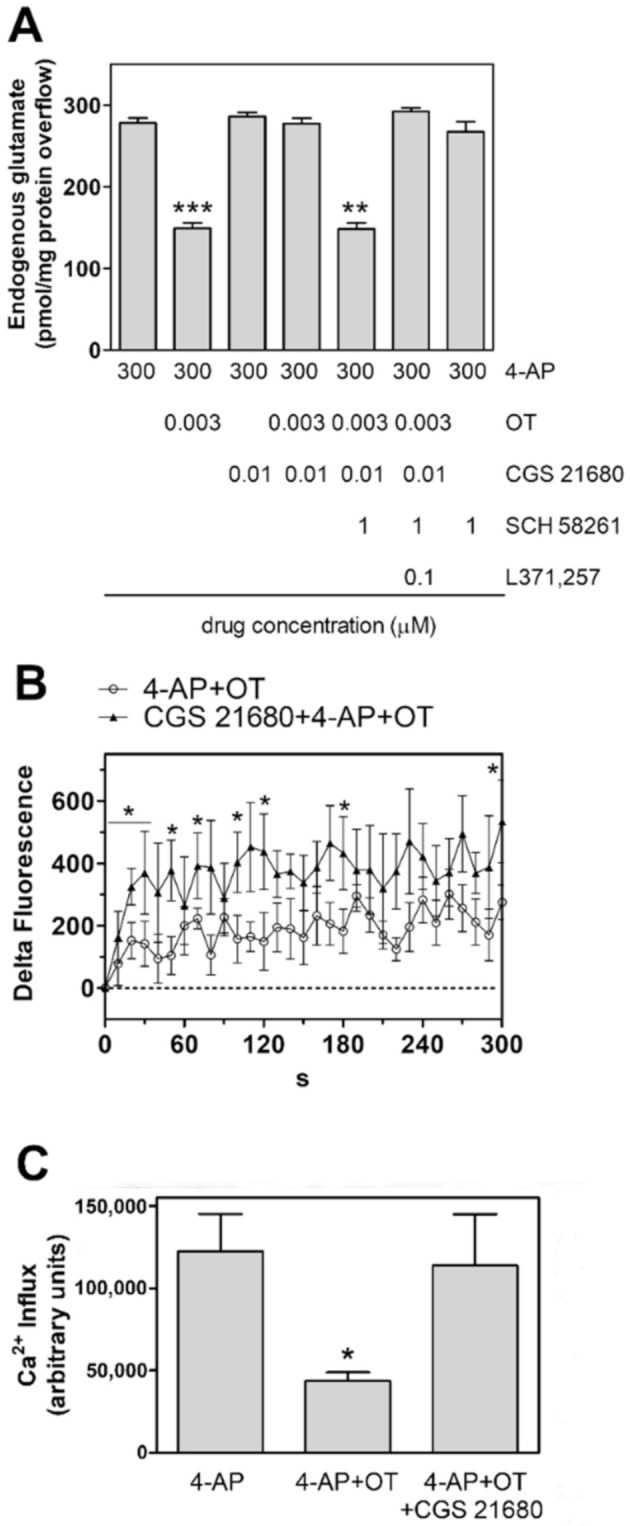
Functional A2A-OT receptor-receptor interaction in striatal gliosomes. (**A**) Inhibition by the A2A receptor agonist CGS 21680 of the OT reduction of 4-AP (300 µM)-evoked efflux of endogenous glutamate release; antagonism by SCH 58261. Bars represent the overflow of glutamate (measured as pmol/mg protein in 3 min sample) in the presence of the drugs at the concentrations indicated. 4-AP was added (6 min) during superfusion; agonists were added together with 4-AP; the antagonists SCH 58261 and L371,257 were added 8 min before the agonists. Other experimental details in Materials and Methods. Data are means ± SEM (bars) of 3–12 independent experiments. *** *p* < 0.0005 compared with the effect of 4-AP; ** *p* < 0.01 compared with the effect of 4-AP in the presence of OT plus the A2A agonist and antagonist, according to Kruskal-Wallis two tailed test and multiple comparison analysis. (**B**,**C**) Inhibition by the A2A receptor agonist CGS 21680 of the OT reduction of 4-AP (300 µM)-evoked increase in [Ca^2+^]_i_, expressed as “Delta Fluorescence” of the CG-dependent fluorescence. The drug concentrations used were the same indicated in (**A**). The areas reported in **C** were quantified to estimate the calcium influxes after 300 s. Data are means ± SEM from 4–6 experiments in duplicate. Other experimental details in Materials and Methods. * *p* < 0.05 vs. 4-AP, according to Mann Whitney (**B**) or Kruskal-Wallis test followed by Dunn’s multiple comparison test (**C**). 4-AP, 4-aminopyridine; OT, oxytocin; CGS21680, 3-(4-(2-((6-amino-9-((2R,3R,4S,5S)-5-(ethylcarbamoyl)-3,4dihydroxytetrahydrofuran-2-yl)-9H-purin-2-yl)amino)ethyl)phenyl)propanoic acid; L371,257, 1-[4-[(1-Acetyl-4piperidinyl)oxy]-2-methoxybenzoyl]-4-(2-oxo-2H-3,1-benzoxazin-1(4H)-yl)piperidine; SCH58261, 7-(2phenylethyl)-5-amino-2-(2-furyl)-pyrazolo-[4,3-e]-1,2,4-triazolo [1,5-c]pyrimidine.

The A2A receptor agonist CGS21680 was ineffective on the glutamate release evoked by 4-AP from the astrocyte processes (Figure 4A). Nevertheless, the A2A receptor agonist CGS21680 (0.01 µM) counteracted the inhibition of the 4AP-evoked glutamate releasing response by 3 nM OT in gliosomes (Figure 4A). The compound SCH 58261 (1 µM), a selective antagonist of the A2A receptors abolished the CGS21680 inhibition of the response to 3 nM OT (Figure 4A). Addition of SCH 58261 at the used concentrations did not affect the 4-AP-evoked endogenous glutamate efflux (Figure 4A). The effect of the A2A agonist was also observed on the OT modulation of the 4AP-evoked increase in [Ca^2+^]_i_ (Figure 4B,C).

Altogether the results indicate a functional interaction between A2A and OT receptors in rat striatal astrocyte processes.

### 2.4. OT and A2A Receptors Expressed on Striatal Astrocytic Process Physically Interact

**Figure 5:**
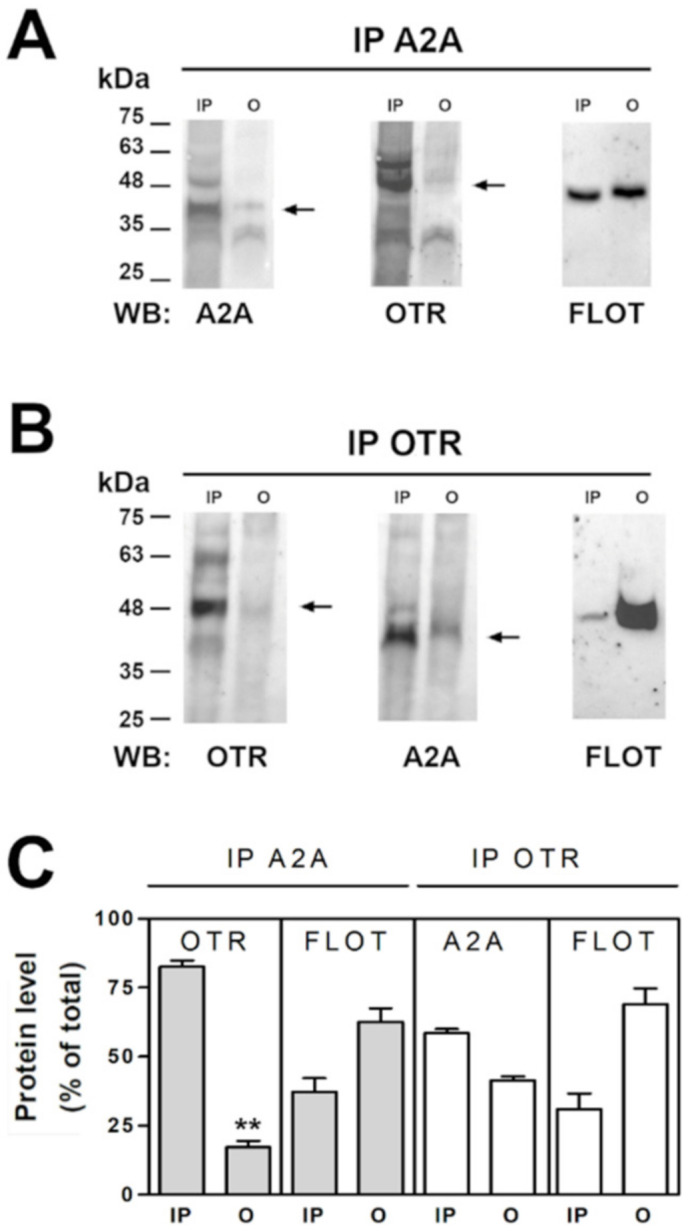
Co-immunoprecipitation of A2A and OT receptors in striatal gliosomes. (**A**) Aliquots (300 µg) of Triton X-100-soluble proteins prepared from striatal fresh isolated gliosomes were immunoprecipitated with 1 µg of anti-A2A antibody (see Material and Methods). The immunoprecipitated (IP) and not immunoprecipitated (O, output) materials were analyzed by immunoblotting using the anti-A2A antibody. IP and O were also analyzed using anti-OTR and the anti-flotillin-1 (FLOT) antibodies. In the figure a representative blot (of three) is shown. A2A, OTR and flotillin-1 immunoreactive bands were quantified and the data were reported in the graph as percentage of the total amount of the relevant protein (% of total). Values are means ± SEM (*n* = 3). (**B**) Aliquots (300 µg) of Triton X-100-soluble proteins obtained from striatal fresh isolated gliosomes were immunoprecipitated with 1 µg of anti-OTR antibody (see Material and methods). IP and O were analyzed by immunoblotting using the anti-OTR antibody. IP and O were also analyzed using anti-A2A receptor and the anti-FLOT antibodies. In the figure a representative blot (of three) is shown. (**C**) Co-immunoprecipitated OTR, A2A and FLOT were quantified and the data were reported as percentage of the total amount of the relevant protein (% of total). Values are means ± SEM (*n* = 3). ** *p* < 0.01 according to Kruskal-Wallis test followed by Dunn’s multiple comparison test.

The capability of A2A and OT receptors on striatal astrocytes to physically interact was investigated by co-immunoprecipitation assay on purified striatal astrocyte processes. By coimmunoprecipitation we found that the A2A and the OT receptors expressed on the striatal astrocytic processes physically interact. Particularly, as shown in Figure 5, by analyzing immunoprecipitated (IP) and not immunoprecipitated (O, output) material by immunoblotting using the anti-OTR and the anti-A2A antibodies we found that the OTR immunoprecipitated together with the A2A receptor (Figure 5A,C) and a fraction of the A2A receptor immunoprecipitated together with the OTR (Figure 5B,C). The findings indicate that the OT receptor expressed on the striatal astrocytic process is associated with the A2A receptor. Moreover, a fraction of flotillin-1, a marker of the membrane lipid rafts [41], coimmunoprecipitated with both the OT and the A2A receptors (Figure 5A–C) suggesting that the receptor complexes were enriched in lipid rafts.

### 2.5. OT and A2A Receptors Expressed on Striatal Astrocytes Can form Heteromers

**Figure 6:**
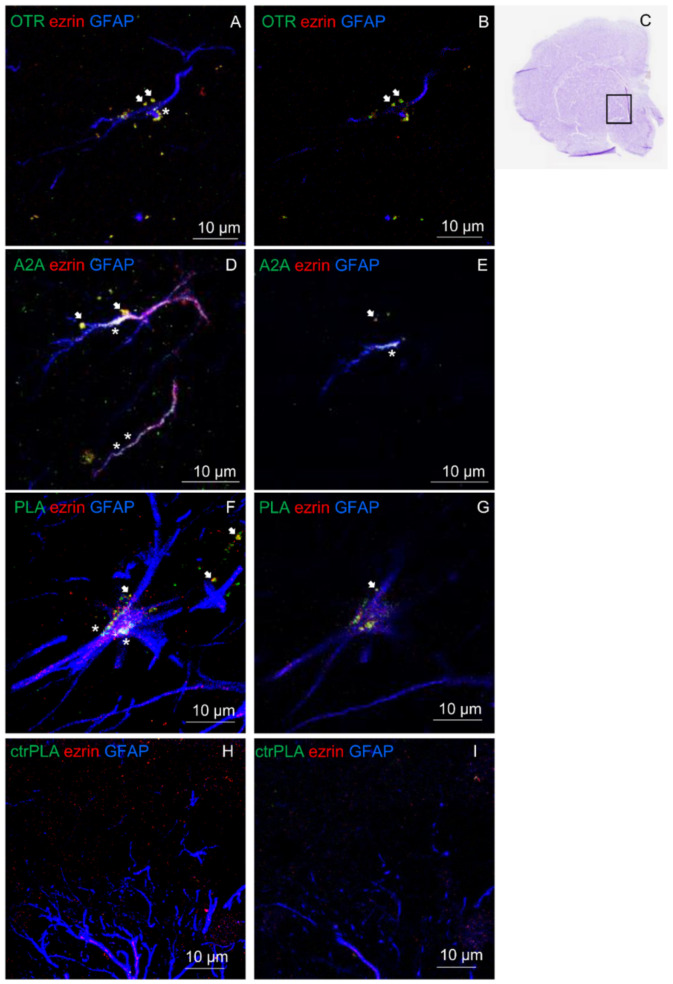
Striatal astrocytes express OT receptors, A2A receptors and A2A-OTR heterodimers. (**A**,**B**,**D**,**E**) The presence of the studied receptors was assessed with immunofluorescence and confocal analysis using primary antibodies (rabbit polyclonal anti-A2A receptor (**D**,**E**), rabbit polyclonal anti-OTR (**A**,**B**), goat polyclonal anti-GFAP and mouse monoclonal anti-ezrin (**A**,**B**,**D**,**E**) in rat hemibrain slices. (**A**,**D**) The merge of the maximum intensity projections of a representative field (240 × 240 µm; z 10 µm) is shown; GFAP (blue), ezrin (red), OTR (green, (**A**,**B**)) or A2A (green, (**D**,**E**)). (**B**,**E**) Merged confocal images of a single z stack of the image shown in (**A**) or (**D**). (**F**,**G**) The detection of in situ Proximity Ligation Assay (PLA) A2A-OTR heteroreceptor complexes was carried out with immunofluorescence and confocal analysis using primary antibodies (mouse monoclonal anti-A2AR, rabbit polyclonal anti-OTR) in rat hemibrain slices. F. The merge of the maximum intensity projections of a representative field (240 × 240 µm; z 10 µm) is shown; GFAP (blue), ezrin (red), PLA for A2A-OTR heteroreceptor complexes appears as green dots. G. Merged confocal images of a single z stack of the image (**F**). (**C**) Cresyl violet staining of the nearest slice used in (**F**). (**H**,**I**) A complete lack of stain for PLA A2A-OTR heteroreceptor complexes was obtained in the negative control experiments, performed avoiding the conjugation of a primary antibody with the Duolink Probes. In the figure the merges of the maximum intensity projections of a representative field ((**H**); 240 × 240 µm; z 10 µm) and a single z stack (**I**) are shown. Scale bars are indicated in the figures. White arrows indicate localization of OTR (**A**,**B**), A2A (**D**,**E**) or PLA dots (**F**,**G**) on ezrin-positive structures. White asterisks indicate localization of OTR (**A**), A2A (**D**,**E**) or PLA dots (**F**) on GFAP-positive structures.

In ventral striatum, astrocytes were identified by the astrocyte markers GFAP and ezrin (see Figure 6A–I). The presence of A2A and OT receptors on striatal astrocytes was assessed by immunofluorescence (Figure 6A–E) while their ability to heteromerize was tested by proximity ligation assay (PLA; Figure 6F,G). The in situ PLA assay showed green spots for A2A-OTR heterodimer complexes in GFAP positive astrocytes and in ezrin-positive fine PAPs as exhibited in a maximum intensity projection in single z stack (Figure 6F,G). As negative controls, we performed the experiment with only one of the two primary antibodies, and PLA signal was not detected (Figure 6H,I). Altogether these data support the existence of A2A-OTR heterodimers in the astrocytes of rat ventral striatum.

### 2.6. Estimated Model of the A2A-OTR Heterodimer

**Figure 7:**
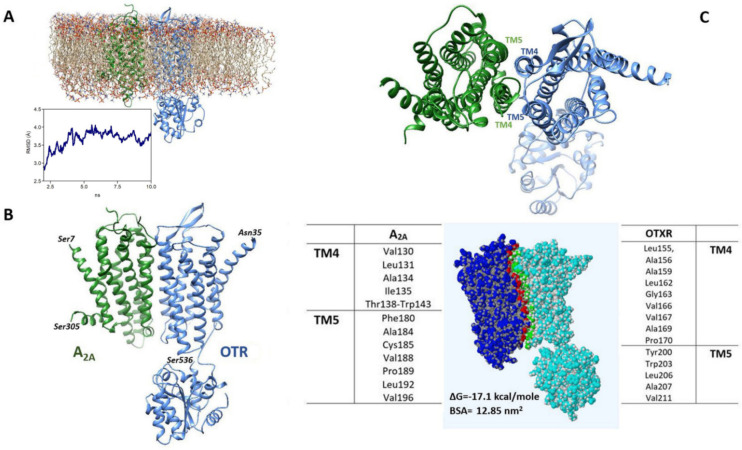
Model of the A2A-OTR heterodimer. (**A**) The heterodimeric structure of A2A and OTR, as obtained by docking, is shown in the environment used for MD, including a POPC lipid bilayer, ions (sodium and chloride, 0.15 M) and water molecules (not shown). The RMSD trajectory during the production phase is also shown. (**B**) Heterodimeric complex between A2A and OTR as predicted by the computational procedure used. (**C**) In the upper panel the extracellular side of the estimated heteromeric structure is shown to indicate the TM4 and TM5 domains. The interface between A2A and OTR is illustrated in the bottom panel together with the residues mainly involved in its formation. The ΔG for complex formation and the interface area (buried surface area, BSA), as predicted by PDBePISA, are also reported.

Molecular Dynamics (MD) simulations in a lipid bilayer of the A2A-OTR dimeric structure estimated by docking methods (Figure 7A) showed that the overall root mean square deviation (RMSD) of the Cα atoms increased at the beginning of the unrestrained phase of the simulation and then stabilized at about 3.7 Å, indicating that the structure reached a stable conformation at 300 °K (shown in Figure 7B). As reported in Figure 7C, the residues predicted to be mainly involved in the heteromerization interface are located in the transmembrane domains 4 and 5 (TM4 and TM5) of both A2A and OTR.

## 3. Discussion

To our knowledge, our study provides the first evidence for the presence and function of OT receptors on striatal astrocytes, and for heteromerization of native OT and A2A receptors in astrocytes. In particular, the following novelties are reported: OTR activation can inhibit the evoked Ca^2+^ signals and release of glutamate from striatal astrocyte processes; both OT and A2A receptors are expressed on the same astrocyte processes in the striatum; astrocytic OT and A2A receptors functionally interact in the control of glutamate release and Ca^2+^ signals; astrocytic OT and A2A receptors can form receptor heteromers. These pieces of evidence were obtained from astrocyte processes acutely prepared from adult rat striatum therefore reflecting the behavior of the processes in mature striatal neuron-astrocyte networks, or on striatal slices from adult rat (PLA).

### 3.1. OTRs Are Expressed on Striatal Astrocyte Processes and Control Ca^2+^ Entry into the Processes and Glutamate Release from the Processes

OTRs were expressed on the astrocyte processes of rat striatum, including the fine PAPs primarily involved in neuron-astrocyte communication at tripartite synapses. Activation of these OTR inhibited the evoked Ca^2+^ signals and release of glutamate from the processes suggesting that astrocytic OTR can control glutamatergic transmission in striatal neuron-astrocyte networks. The inhibitory effects of OT on the evoked Ca^2+^ signals and release of glutamate from the processes deserve to be commented. In fact, while most works focused on excitatory OT actions through OTRs coupled to Gq and IP3 signalling, with increased intracellular Ca^2+^ and neuronal excitation, accumulating evidence indicates that OT can also act through different intracellular pathways. Indeed, OT was reported to depress the evoked excitatory post-synaptic currents through presynaptic OTRs [42], to reduce the depolarization-evoked Ca^2+^ response [43] or to inhibit glutamate release by modulating voltage-operated Ca^2+^ channels [44]. Interestingly, some OTR ligands can behave as “biased agonist”, acting as an agonist for Gi-coupled OTRs, but as an antagonist for Gq-coupled OTRs, or selectively activate the Gi or the Gq pathway [7,45]. These results suggest that OT modulation and plasticity may not be simply related to a single facilitatory mechanism, and doesn’t involve only Gq protein mediated signalling. Additionally, the OT concentration may be relevant; in many papers OT was used at 1 µM concentration or over, much higher than the concentration we used in our experimental conditions. Indeed, the narrow concentration dependency of OT effect in our conditions is puzzling. Nevertheless, the same OT concentration (3 nM) was already described able to interact with the dopamine D2 receptor and to increase the D2 receptor/Gi/o coupling while lower or higher concentrations were ineffective [11].

In apparent contradiction with our results, OT was reported to evoke Ca^2+^ transients in amygdala astrocytes [3] as well as in cultured hypothalamic or hippocampal astrocytes [46,47,48,49,50]. However, these reports refer to astrocyte somata, while here we investigate OT effects on Ca^2+^ microdomains at the astrocytic processes. As a matter of fact, astrocytic Ca^2+^ signals are differently regulated in different subcellular regions: while Ca^2+^ transients in the somata are mainly related to Ca^2+^ release from intracellular stores, Ca^2+^ entry through channels or ionotropic receptor channels significantly contribute to Ca^2+^ signals at processes/PAPs [51,52,53]. As far as the inhibition of glutamate release is concerned, to our knowledge, this is the first report indicating a direct OT modulation of glutamate release from astrocytes. Indeed, in the hypothalamic supraoptic nucleus or in the amygdala evidence is available indicating that, by evoking release of serine from astrocytes [3,18] or by facilitating glutamate diffusion from the synapses [14,15,16], OT can indirectly facilitate glutamate transmission, while to our knowledge direct effects of OT on glutamate release from astrocytes have never been reported. Conversely, OT inhibition of neurotransmitter release from nerve terminals (specifically of glutamate or GABA) related to inhibition of Ca^2+^ entry into the terminals, has been reported [44,53,54]. In particular, the inhibition of the 4-AP depolarization evoked glutamate release from the striatal astrocyte processes by OT fits in well with the OT inhibition of depolarization-evoked Ca^2+^ entry into the processes. In point of fact, 4-AP was found to evoke exocytotic vesicular release of glutamate from striatal astrocyte processes, abolished in the absence of extracellular Ca^2+^ [40], consistent with ultrastructural evidence for VGLUT in the processes [55] or in striatal astrocytes in situ [56], and with the ability of in situ striatal astrocytes to release glutamate as a consequence of an increase of [Ca^2+^]_i_ levels [38]. The ability of OT to modulate the evoked release of glutamate from the striatal astrocyte processes add a new complexity to the scenario of OT modulatory effects on the central nervous system, indicating that the OT regulation of glutamatergic transmission can operate at multiple levels.

### 3.2. A2A and OT Receptors Are Expressed on the Same Astrocyte Processes and Interact to Control Glutamate Release and Ca^2+^ Signals

As shown by confocal imaging, both A2A and OTR were expressed on the same astrocyte processes, opening the possibility of direct receptor-receptor interactions at the plasma membrane of the processes. Activation of A2A receptor—per se unable to affect the glutamate release or intracellular Ca^2+^ levels in striatal processes—abolished the OT inhibition of glutamate release and Ca^2+^ signals in the processes, demonstrating a RRI through which OT and A2A receptors could regulate glutamatergic transmission in striatal neuron-astrocyte networks. We previously found that A2A receptors can also dampen the D2 receptor-mediated inhibition of glutamate release from striatal astrocyte processes [40,54], suggesting multiple roles of astrocytic A2A receptors in the control of striatal glutamatergic transmission.

### 3.3. Astrocytic A2A and OT Receptors form Receptor Heteromers

To our knowledge, the ability of A2A and OT receptors to interact by heteromerization has never been investigated. Our findings indicate that native OT and A2A receptors expressed on striatal astrocyte processes can undergo RRI based on receptor heteromerization. Co-immunoprecipitation of the receptors indicates that the RRI was based on a physical interaction. Above all, while the OTR almost completely immunoprecipitated together with the A2A receptor, only a fraction of the A2A receptor coimmunoprecipitate together with the OTR, suggesting that at striatal astrocyte processes the A2A receptors may remain as monomers, or possibly behave as a hub receptor which interacts also with other receptor types. Co-immunoprecipitation of OT and A2A receptors with the membrane lipid rafts marker flotillin-1 [41] suggests enrichment of the receptor complexes in lipid rafts, which may provide the ordered membrane microenvironment for horizontal molecular networks of GPCRs complexes [57,58] in the astrocytic processes membrane. PLA analysis confirmed that OT and A2A receptors form heteromers on GFAP-positive striatal astrocytes and on the ezrin-positive fine processes. It is to note that ezrin is involved in the structural changes related to astrocyte activation, required for PAPs motility and regulation of synapse coverage [59,60]. The presence of OTR and A2A-OTR heteromers on ezrin-positive fine striatal astrocyte processes might suggest the OT involvement in the control of astrocytic coverage of the synapses, therefore in the balance and integration of wiring and volume transmission at striatal level too.

The estimated model of the A2A-OTR heterodimer indicated that the structure could reach a stable conformation and predicted that residues located in the TM4 and TM5 of both A2A and OT receptor may be mainly involved in the heteromerization interface. In particular, the transmembrane domain TM5 of the A2A receptor was found able to form part of the heteromerization interface with the TM4 of the D2 receptor in cells [61,62,63]. We previously reported that in the same preparation of striatal astrocyte processes native A2A receptor can heteromerize with the D2 receptor [40,55,64]. It remains to be clarified if the A2A receptors may also form higher level heteromers and by which mechanisms at the level of striatal astrocytes.

## 4. Materials and Methods

### 4.1. Animals

Adult male rats (Sprague Dawley 200–250 g), bred at the animal care facility of Department of Pharmacy (DIFAR), University of Genova, Italy, were housed at constant temperature (22 ± 1 °C) and relative humidity (50%), under a light–dark schedule (lights on 7 AM–7 PM), and with free access to standard pellet diet and water. The experimental procedures and animal care complied with the European Communities Parliament and Council Directive of 22 September 2010 (2010/63/EU) and with the Italian D.L. n. 26/2014, and were approved by the Italian Ministry of Health (protocol number 30/11/2016-OPBA of November 2016), in accordance with Decreto Ministeriale 116/1992. All possible efforts were made to minimize animal suffering and the number of animals used per experiment.

### 4.2. Preparation of Purified Astrocytic Processes

The striatum was rapidly removed after decapitation, and placed in ice-cold medium. Purified astrocyte processes (gliosomes) were prepared according to Nakamura et al. (1993) [65], as previously reported [40,55,66,67,68]. Briefly, the tissue was homogenized with a glass-Teflon tissue grinder (clearance 0.25 mm) in 10 volumes of 10 mM Tris/HCl pH 7.4 containing 0.32 M sucrose. After centrifugation of the homogenate (5 min at 1000× *g*; 4 °C) to remove nuclei and debris, the supernatant was stratified on a discontinuous Percoll gradient (2, 6, 10 and 20% (*v*/*v*) in Tris-buffered sucrose) and centrifuged (5 min at 33,500× *g*; 4 °C). The layer between 2% and 6% (*v*/*v*) Percoll (gliosomes; purified astrocyte processes) was collected and washed by centrifugation. Gliosomes are a preparation of astrocytic processes with negligible neuronal contamination, containing gliotransmitter-loaded vesicles and competent for gliotransmitter secretion [67]. For release experiments or [Ca^2+^]_i_ assay, purified astrocyte processes were suspended in standard HEPES medium (mM: NaCl 128, KCl 2.4, MgSO_4_ 1.2, KH_2_PO_4_ 1.2, CaCl_2_ 1.0, and HEPES 10 with glucose 10, pH 7.4). For immunofluorescence confocal microscopy, gliosomes were resuspended in standard HEPES medium at 1 μg proteins/μL. Protein determinations were carried out according to Bradford [69].

Striatal gliosomes have proven to be positive for the astrocyte marker GFAP and for ezrin, a marker that identifies the astrocyte processes, and negative for synaptophysin, integrin-αM, and RIP, which are markers for nerve terminals, microglia, and oligodendrocytes, respectively, indicating that gliosomes are a purified preparation of processes of striatal astrocytes, negligibly contaminated by neuronal, microglial or oligodendroglial particles [40], confirming the evidences obtained in cerebellar or cerebrocortical preparations [67,70].

### 4.3. Endogenous Glutamate Release

Release of the gliotransmitter glutamate from the astrocyte processes was studied by applying to the processes (gliosomes) the method for measuring neurotransmitter release from nerve terminals in superfusion. Notably, monitoring the release of a gliotransmitter from a superfused gliosomal monolayer (or the release of a neurotransmitter from a superfused synaptosomal monolayer [71], when superfusion removes any possibly released active substance and avoids formation of receptor biophase, allows exposure of “nude” receptors. Thus, it can be employed as a simplified model for the pharmacological characterization of release regulating receptors as well as for the analysis of receptor-receptor interactions [40,55,72,73]. Briefly, gliosomes were transferred to parallel superfusion chambers and superfused with standard medium (0.5 mL/min; 37 °C). After 33-min superfusion, superfusate fractions were collected in 3min samples (from the first basal fraction, B1, to B5); after 38 min superfusion, gliosomes were exposed to depolarizing stimulus (300 µM 4-AP; 6 min). The A2A agonist and/or OT were added together with 4-AP; the effect of antagonists was evaluated by adding the compounds 8 min before the agonist. In each experiment at least one chamber, superfused with standard medium or with medium appropriately modified, was used as a control for each condition. The amount of endogenous glutamate released in the fractions collected was measured by high-performance liquid chromatography, as previously described [74]. The analytical method involved automatic precolumn derivatization (Waters Alliance; Milford, MA, USA) with o-phthalaldehyde, followed by separation on a C18 reverse-phase chromatography column (Chrompack International, 10 cm 4.6 mm, 3 mm) and fluorimetric detection. Homoserine was used as an internal standard. The detection limit was 100 fmol/mL. Protein determinations were carried out according to Bradford [69]. The amount of endogenous glutamate released in the fractions was expressed as pmol/mg protein. The mean endogenous glutamate release in B1 and B2 fractions was taken as the 100% control value for each chamber; endogenous glutamate efflux in Bn fractions was evaluated as the percent variation with respect to the corresponding control value. The drug-evoked endogenous glutamate efflux was measured by subtracting the area under the curve of percent variations in endogenous glutamate release in appropriate control chambers from the area under the curve of the percent variations in drug-treated chambers.

### 4.4. [Ca^2+^]_i_ Assay

[Ca^2+^]_i_ was determined as previously described [68,75]. Briefly purified astrocyte processes were washed once in HEPES buffer and then incubated in the same buffer containing 10 μM Calcium Green™-1 AM (CG). After 30 min at 37 °C, synaptosomes and gliosomes were washed twice with HEPES buffer, transferred to a black 96-well microplate (50 µg/well), and then exposed to drugs. The fluorescence intensity (excitation 485 nm and emission 535 nm) was measured every 10 s for 5 min using the top reading mode in the fluorescence multilabel reader LB 940 Mithras (Berthold Technologies, Baden Württemberg, Germany). [Ca^2+^]_i_ increase is expressed as “Delta Fluorescence”, which is the difference between the CG-dependent fluorescence of the stimulated samples and the ones of the vehicle-treated samples, both measured at each recording time and subtracted by the one measured at the starting time.

### 4.5. Immunofluorescent Confocal Microscopy

Immunofluorescent confocal microscopy was carried out as previously described [40,55]. Gliosomes (15–20 µg) were fixed with 2% paraformaldehyde, permeabilized with 0.05% Triton X-100 (5 min) and incubated 60 min with primary antibodies diluted in phosphate buffer saline (PBS) containing 3% albumin. The following antibodies were used: goat anti-GFAP (1:500; Santa Cruz Biotechnoloy Inc., Dallas, TX, USA); mouse anti-A2A receptor (1:200; Merck Millipore Corporation, Milan, Italy); rabbit anti-OTR (1:200; Alomone Labs, Jerusalem, Israel); mouse anti-ezrin (1:100; Sigma-Aldrich, Milan, Italy) and guinea pig anti-vesicular glutamate transporter 1 (VGLUT1; 1:500; Merck Millipore Corporation). After washing with PBS, the preparations were incubated (60 min) with Alexa Fluor 488, 546 or 633 secondary antibodies conjugates (1:1000; Life Technologies Corporation, Carlsbad, CA, USA) in PBS containing 0.5% albumin. Images were collected by confocal microscopy using a three-channel TCS SP2 laser-scanning confocal microscope (Leica, Wetzlar, Germany), equipped with 458, 476, 488, 514, 543 and 633 nm excitation lines. Spatial colocalization was analyzed through two-dimensional correlation cytofluorograms obtained by means of macro routines integrated as plug-ins in ImageJ Fiji software (Wayne Rasband, National Institutes of Health, Bethesda, MD, USA). Red and green or blue labels were considered as colocalized in the same pixel if their respective intensities (0–255, eight bit) were strictly higher than the threshold of their channels, as determined by analyzing the color histograms. Data were collected from 10–12 fields from three different preparations, and are expressed as mean ± SEM.

### 4.6. Immunoprecipitation and Immunoblot

Gliosomes obtained from striata of four animals were suspended in 50 mM sodium borate buffer, pH 7.5 with 1 mM EDTA and Protease Inhibitor Cocktail (lysis buffer) at 1 mg/mL and lysed by three cycles of freezing and thawing followed by sonication. Protein quantification of lysate was performed using the Bradford method [66]. To perform the immunoprecipitation, gliosomes lysate was centrifuged at 100,000× *g* for 30 min at 4 °C. The pellet was washed once and then solubilized in 100 µL of 50 mM sodium borate, 0.1 mM EDTA, pH 7.5 (immunoprecipitation buffer) + 1% Triton-X at 37 °C for 1 h. Then 400 µL of immunoprecipitation buffer have been added to lysate to dilute the Triton-X to 0.2% (Total of the membranes) and then centrifuged at 18,000× *g* for 15 min at 4 °C. The supernatant has been precleared with protein G-sepharose, and then incubated in the presence of 1µg of anti-A2A receptor antibody or anti-OTR antibody at 4 °C, overnight. Protein G-sepharose was then added to the sample and incubated for an additional 1h at room temperature (RT). The immunocomplexes were centrifuged at 400× *g* and aliquots of supernatant were submitted to SDS-PAGE. The immunocomplexes were washed three times with immunoprecipitation buffer + 0.1% Triton-X, heated in SDS-PAGE loading buffer for 5 min and submitted to 10% SDS-PAGE followed by electroblotting onto a nitrocellulose membrane and saturated with phosphate-buffered saline, pH 7.5, containing 5% skim milk powder. The blots were probed with specific antibodies and the immunoreactive material was detected with a Bio-Rad Chemi Doc XRS apparatus.

### 4.7. Preparation of Striatal Slices and Cresyl Violet Staining

After decapitation, the brain was rapidly removed, and processed to prepare cryostat sections, essentially as previously described [76]. Briefly, the left/right half of the brain was embedded in killik (Bio-Optica, Milan, Italy), frozen in liquid nitrogen and sectioned using a cryostat (Leica CM1900UV, Wetzlar, Germany). 10–15 µm-thick coronal sections were collected on poly(L)-lysine-coated slides and stored at −20 °C until further processing. Selected sections were thawed at RT, stained with cresyl violet (Bio-Optica, Milan, Italy), differentiated in two changes of 95% ethanol, quickly dehydrated with absolute ethanol, cleared with Bio-clear (Bio-Optica, Milan, Italy) and permanently mounted with Eukitt (Bio-Optica, Milan, Italy). Whole-slide images were acquired using the Manual WSI software (Microvisioneer, Esslingen am Neckar, Germany) and a BX60 microscope (Olympus, Hamburg, Germany) equipped with a color camera (Basler, Ahrensburg, Germany).

### 4.8. Proximity Ligation Assay

Proximity ligation assay (PLA) was carried out essentially a described in studies conducted by Trifilieff et al. (2011) [77] and by Pelassa et al. (2019) [64]. In situ PLA was performed on 10–15 µm rat emi brain slices using the primary antibodies (mouse anti-A2A receptor (1:200, Merck Millipore Corporation); rabbit anti-OTR (1:200; Alomone Labs)), and the Duolink in situ PLA detection kit (DUO92014, Sigma-Aldrich). PLA was performed according to the manufacturer’s instructions using the Duolink Detection Kit (DUO92014, DUO92002, DUO92004 Sigma-Aldrich). Briefly, slices were first washed three times for 5 min in PBS, then blocked and permeabilized in Blocking Solution, as indicated in the kit instruction. The slices were incubated with the anti-A2A and anti-OTR antibodies diluted in Antibody Diluent solution in a humid chamber over-night at 4 °C. Thereafter, the sections were rinsed with Wash Buffer A 1X and incubated with species-specific secondary antibodies conjugated to complementary oligonucleotides for 1 h at 37 °C (DUO92002, DUO92004 Sigma-Aldrich). After hybridization, ligation and amplification steps were performed using manufacturer’s instruction and fluorescence images were acquired enabling the visualization of the A2A-OTR heteromer by green fluorescent at confocal microscopy.

For GFAP and ezrin colocalization analysis after the amplification step the slices were rinsed in Wash Buffer B 1X, incubated with goat anti-GFAP (1:500, Santa Cruz Biotechnoloy Inc.) and mouse anti-ezrin (1:100; Sigma-Aldrich) in Antibody Diluent solution in a humid chamber over-night at 4 °C and subsequently with Alexa Fluor 633-conjugated donkey anti-goat and 546 donkey anti-mouse for 1h at RT. Finally, the slices were rinsed in Wash Buffer B 0.01X, mounted in Mounting Medium solution (DUO82940) and examined under a confocal laser scanning microscope. Negative control experiments were conducted avoiding the conjugation of the primary anti-A2A or anti-OTR antibody with the Duolink Probes and resulted in a complete lack of stain for PLA. The specificity of the double immunolabeling was verified by replacing the primary antibodies with PBS (see also [64]).

The immunofluorescence measurements were performed using a Leica STELLARIS 8 Falcon τSTED (Leica Microsystems, Mannheim, Germany) inverted confocal/STED microscope. Excitation was provided by a white light laser selecting the combination of chosen fluorochromes to avoid crosstalk. Detection has been performed by three Power HyD detectors. The fluorescence image (1024 × 1024 × 32 bit) acquisition was performed using an HC PL APO CS oil immersion objective 100× (1.40 NA). The pinhole was set to 1 Airy size. Line scanning speed ranged was 400 Hz. Leica “LAS X application Suite” software package was used for acquisition, storage, visualization, and 3D analysis.

### 4.9. Receptor Structures

Experimentally assessed molecular structures of human adenosine A2A receptor and OTR were retrieved from the Protein Data Bank (https://www.rcsb.org, accessed on 7 September 2021). Both A2A (PDB code: 3PWH [78]) and OTR (PDB code: 6TPK [79]) were experimentally obtained by X-ray diffraction at a resolution of 3.30 Å and 3.20 Å respectively.

By using the DockPrep module available in the UCSF Chimera molecular modeling software (Resource for Biocomputing, Visualization, and Informatics, University of California, San Francisco, CA, USA; http://www.rbvi.ucsf.edu/chimera, accessed on 8 September 2021) all extra molecules (such as ligands) were removed, hydrogens were added and partial charge assigned. The obtained molecular structures were then energy minimized by using the Yasara software (http://www.yasara.org/minimizationserver.htm, accessed on 8 September 2021 [80]) and stored for further processing.

### 4.10. Modeling the A2A-OT Heteroreceptor Complex

Structures of possible heterodimers of A2A and OT receptors were estimated following a previously reported procedure [81]. Briefly, the GalaxyHeteromer (http://galaxy.seoklab.org/, accessed on 10 September 2021) software was used to perform protein-protein docking [82] and after ranking the solutions by energy score, the best solution exhibiting the correct orientation of the monomers was selected as a possible heterodimeric structure. To refine the obtained heterodimer using a more realistic model of the biological environment, the structure was inserted into a pre-equilibrated 1-palmitoyl-2oleoylphosphatidylcholine (POPC) bilayer (see Figure 7A) using CHARMM-GUI Membrane Builder (http://www.charmm-gui.org/?doc=input, accessed on 11 September 2021), a simulation preparation software [83]. Molecular Dynamics simulations were then run (see [81]) using the NAMD package [84] (version 2.12) with the CHARMM36 force field [85], powered by the VMD software [86] for data visualization and management. The system first underwent 1 ns equilibration phase, involving heating from 0 °K to 300 °K, followed by a production (unrestrained) phase of 10 ns. The protein complex configuration at the end of the production phase was taken as predictive of the heterodimer structure and the structural features of the predicted heteromerization interface were explored by the PDBePISA tool [87] freely available at https://www.ebi.ac.uk/pdbe/pisa/, accessed on 30 September 2021.

### 4.11. Calculations and Statistical Analysis

Means ± SEM of the numbers of experiments (*n*) are indicated throughout. Significance of the difference was analyzed by the non-parametric Kruskal-Wallis two tailed test and multiple comparison analysis and Mann-Whitney test, with statistical significance being taken at *p* < 0.05. Statistical analysis as well as the determination of the areas underlying CG-dependent curves were carried out using the Prism 4.02 software package (GraphPad Software, San Diego, CA, USA).

### 4.12. Materials

4-Aminopyridine (4-AP), Triton-X 100, oxytocin (OT) and SCH 58261 were purchased from Sigma-Aldrich, while L371,257 were from Tocris. When possible, drugs were dissolved in distilled water or in physiological medium. SCH 58261 was dissolved in DMSO and then diluted 1:1000 in physiological medium. DMSO 0.1% had no effect on endogenous glutamate release from gliosomes. All the salts were from Sigma-Aldrich. Protein G-sepharose 4 Fast Flow, nitrocellulose membrane and ECL Select were obtained from GE Healthcare, Milan, Italy. The anti-rabbit secondary antibody and the Protease Inhibitor Cocktail were obtained from Cell Signaling Technology, Danvers, MA, USA.

## 5. Conclusions

OT is recognized to function as a reward signal for affiliative interactions, playing a key role in adaptive processes associated with reward, tolerance, memory and stress responses (see [8] and references therein); indeed, blockade or ablation of OT receptors in the nucleus accumbens was found to impair social reward processing [88,89]. In reality, animal models indicate that OTRs in the ventral striatum modulate sensitivity to social reinforcers, social behavior and emotional states (see [13] and references therein). In humans, a study aimed to investigate OT-dependent brain mechanisms associated with social-affective problems in adolescents, showed that OTR genotype might modulate the ventral striatum activity and responsiveness to negative social-emotional cues, being related to social/emotional problems and resilience against stressful life events [13].

It has been proposed that differences in the endogenous oxytocinergic system may affect susceptibility to drug addiction [90], and evidences are accumulating indicating that OT can be involved in the control of drug abuse [8]. The OT ability to reduce drug-seeking and drug-induced behaviors has been repeatedly reported [8], although the mechanisms involved are not completely understood. Recently, the idea that direct effects of OT on glutamatergic transmission within the reward processing pathway, including the ventral tegmental area and the ventral striatum, may contribute to OT therapeutic potential against substance use disorder has been put forward [91]. Actually, glutamate seems to be involved in positive reinforcing and drug reward, and altered glutamatergic transmission in the ventral striatum could mediate the rewarding effects of drugs of abuse [92] and relapse in drug addiction [93]. According to the glutamate homeostasis hypothesis of substance use disorder, alterations of striatal astrocyte function may have implications in addiction: imbalance in glial regulation of glutamatergic transmission at prefrontal-to-accumbens synapses in chronic drug use may lead to drug seeking and relapse [94] and reinstatement vulnerability [95]. The importance of astrocytes, and how they contribute to glutamatergic dysregulations in ventral striatum in drug addiction has been recently highlighted, and argued to provide potential targets for therapeutic approaches to reward dysfunction and addiction, and aid strategies for drug development to treat substance use disorders [39,95,96]. Notably, OT has been proposed to reduce addictive behaviors by restoring the drug-induced changes in the glutamatergic transmission and in the astrocyte function in ventral striatum [91].

It is also worth mentioning the ability of A2A receptor to influence the reinforcement processes underlying ethanol, opiate and psychostimulant intake. Adenosine, mainly through A2A receptors, was reported to play a facilitative role in the mediation of the effects of opiates, of the opiate rewarding effects of withdrawal [97]. As a matter of fact, A2A receptor antagonists have long been proposed to become therapeutic agents for drug addiction [98]. The ability of A2A to heteromerize with OTRs has to be added to the evidence for A2A heteromerization with D2 receptors on striatal astrocytes [40,55,64] and with multiple neurotransmitter receptors on glutamatergic or enkephalinergic/dynorphinergic/GABAergic striatal input [96,97], all this towards a better comprehension of the complex scenario of A2A control of glutamatergic transmission in the striatum, and of the apparently conflicting results on A2A receptors as target for therapeutic approaches to substance use disorder [99,100].

The evidences here reported, putting together OT (and A2A) receptors, striatal astrocytes and glutamate release, may contribute to shed light on how oxytocinergic system alterations may affect astrocyte function and lead to glutamatergic dysregulation in striatum. A better knowledge of the OT ability to influence the efficacy of glutamatergic transmission in striatum in physiological conditions or in glutamate transmission dysfunction, may help to understand the OT therapeutic potential in drug addiction. In particular, our findings are consistent with the proposal of signaling through GPCR heteromers [101,102], and of astrocytes as pharmacological target for neuropsychiatric disorders, and suggest that striatal astrocytic OTRs and A2A-OT heteromers, might represent a target for tuning of striatal glutamatergic transmission potentially useful in drug addiction.

## Data Availability

Data available on request from the corresponding authors.

## Supplementary Materials

The following supporting information can be downloaded at: https://www.mdpi.com/article/10.3390/ijms23042326/s1.
