# Supplementary material for: Heterodimer of A2A and Oxytocin Receptors Regulating Glutamate Release in Adult Striatal Astrocytes
Source: Int J Mol Sci. 2022 Feb 19;23(4):2326. doi: 10.3390/ijms23042326 (PMC8879615; doi:10.3390/ijms23042326)
Supplement: Supplementary file 1 [file ijms-23-02326-s001.zip › ijms-1583588-supplementary.pdf]

Supplementary material

Heterodimer of A2A and Oxytocin Receptors Regulating Glutamate Release in Adult Striatal Astrocytes

Sarah Amato [1], Monica Averna [2], Diego Guidolin [3], Marco Pedrazzi [2], Simone Pelassa [1,†], Michela Capraro [2], Mario Passalacqua [2,4], Matteo Bozzo [5], Elena Gatta [6], Deanna Anderlini [7], Guido Maura [1], Luigi F. Agnati [8], Chiara Cervetto [1,*] and Manuela Marcoli [1,9,*]

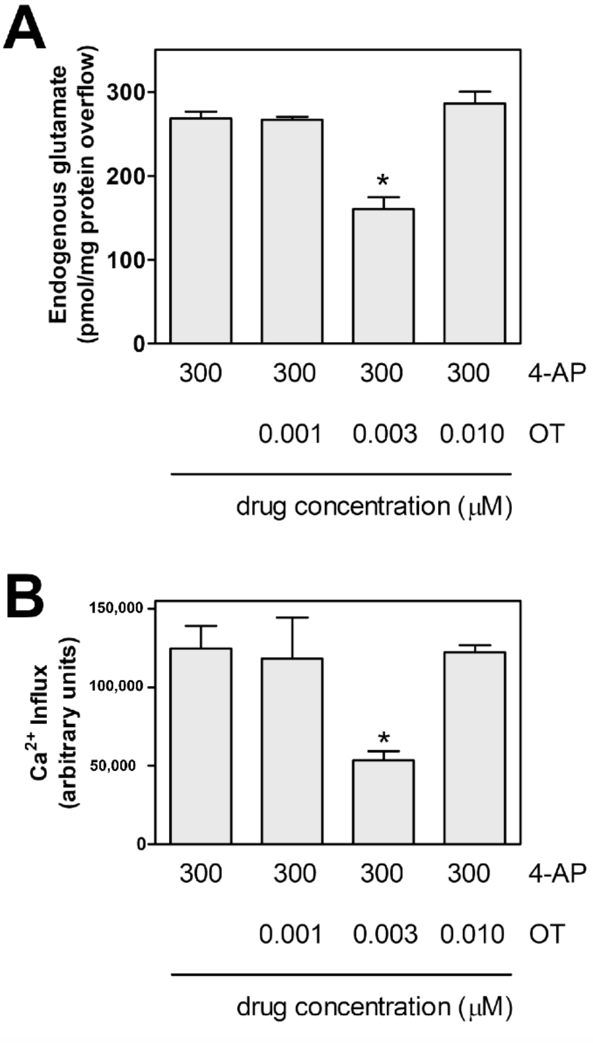

Figure S1. Endogenous glutamate release and intracellular calcium increase in response to 4-AP induced depolarization in striatal gliosomes. Modulation by OT at different concentrations. **A**. Inhibitory effect of OT 3 nM on the 4-AP-evoked glutamate release; ineffectiveness of OT 1 nM or 10 nM. Bars represent the percent variation of the glutamate release in the presence of the drugs at the concentrations indicated. 4-AP was added (6 min) during superfusion; OT was added together with 4-AP. Other experimental details in Materials and Methods. Data are means ± SEM (bars) of n= 3-6 independent experiments. * p<0,005 compared with the effect of 4-AP, according to Mann Whitney test. **B.** CG-loaded gliosomes: inhibitory effect of OT 3 nM on the 4-AP-evoked calcium influx; ineffectiveness of OT 1 nM or 10 nM. The 4-AP-evoked increase in $[Ca^{2+}]_i$ was expressed as "Delta Fluorescence" of the CG-dependent fluorescence Bars represent the calcium influx after 300 s in the presence of the drugs at the concentrations indicated. The areas were quantified to estimate the calcium influxes after 300 s. Other experimental details in Materials and Methods. Data are means ± SEM from five (4-AP), six (4-AP + OT 3 nM), or three experiments in duplicate. * p<0.05 compared with the effect of 4-AP, according to Kruskal-Wallis test followed by Dunn's multiple comparison test. 4-AP, 4-aminopyridine; CG, Calcium Green™-1 AM; OT, oxytocin.
